# Harnessing Plant Biodiversity for the Discovery of Novel Anticancer Drugs Targeting Microtubules

**DOI:** 10.3389/fpls.2017.00720

**Published:** 2017-05-04

**Authors:** Songbo Xie, Jun Zhou

**Affiliations:** Key Laboratory of Animal Resistance Biology of Shandong Province, Institute of Biomedical Sciences, College of Life Sciences, Shandong Normal UniversityJinan, China

**Keywords:** microtubule, microtubule-targeting agent, plant, cancer chemotherapy, drug discovery

## Abstract

The microtubule cytoskeleton plays a critical role in a wide range of cellular activities and has been shown to be a highly effective target for the treatment of human malignancies. Despite the recent focus on proteomics and high-throughput profiling, it is clear that analysis of plant extracts has yielded several highly efficacious microtubule-targeting agents (MTAs) currently in clinical use, as well as agents in the current pipeline with promising efficacy. To date, a large proportion of the world’s plant biodiversity remains untapped by the pharmaceutical industry, presenting a major opportunity for the discovery of novel pharmacologically active lead compounds. Because plants contain an astonishing array of structurally diverse molecules, they represent an ideal source for the discovery of novel MTA leads. To demonstrate the importance of searching for novel bioactive compounds across the plant kingdom, herein, we summarize the discovery and development of plant-derived MTAs and discuss the challenges associated with searching for novel bioactive compounds from plants. We propose potential solutions to these problems with the aim of facilitating further exploration and identification of novel MTAs from plant biodiversity.

## Introduction

Historically, plants have served as important sources of medicinal products for the treatment of human malignancies (Fridlender et al., 2015; Cragg and Pezzuto, 2016). Renowned examples include paclitaxel, vincristine, and vinblastine, all of which target microtubules. Microtubules are evolutionarily conserved cytoskeletal components present in nearly all eukaryotic cells, and they play vital roles in diverse cellular activities, such as cell division, cell migration, and intracellular transport (Akhmanova and Steinmetz, 2015). Microtubule-targeting agents (MTAs) are able to block cell division and induce apoptosis, a property critical for their clinical utility as anticancer drugs. In this review, we examine the key steps in the development of plant-derived MTAs and highlight plant biodiversity as an important opportunity for the discovery of novel MTAs with anticancer activities.

## Microtubule Structure and Function

**FIGURE 1:**
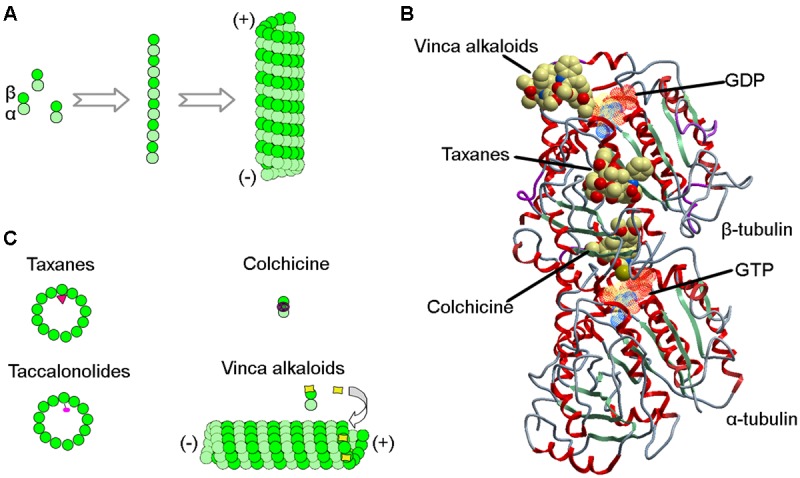
**Interactions of MTAs with microtubules. (A)** During microtubule polymerization, α/β-tubulin heterodimers assemble head-to-tail to generate protofilaments, which form lateral connections to build the hollow structure of the microtubule. **(B)** Structure of the α/β-tubulin heterodimer showing binding sites for taxanes, vinca alkaloids, colchicine, GDP, and GTP. **(C)** Schematics showing how taxanes, vinca alkaloids, colchicine, and taccalonolides are incorporated into microtubules or α/β-tubulin heterodimers.

Microtubules are hollow, cylindrical polymers assembled from α- and β-tubulin heterodimers in a head-to-tail manner. The polymerization of cellular microtubules is initiated by the formation of a primer consisting of the γ-tubulin ring complex, a process termed nucleation. The α/β-tubulin dimers then use this structure as a template for polymerization, forming linear protofilaments that subsequently make lateral associations to generate sheets, and eventually polarized, hollow cylinders (**Figure 1A**). In animal cells, microtubules mainly extend from the centrosome, forming a hub-and-spoke-like array. The minus ends of microtubules are anchored in the centrosome, while the plus ends face the plasma membrane (Conduit et al., 2015).

Microtubules undergo frequent transitions between growth and shortening phases, which endows microtubules with dynamic instability, a property critical for most of the microtubule functions (Akhmanova and Steinmetz, 2015; Ohi and Zanic, 2016). During mitosis, microtubules are more dynamic and undergo dramatic reorganization to assist in the formation of the bipolar mitotic spindle and segregation of chromosomes. Chemical agents that interfere with microtubules impair the assembly of the spindle apparatus, arresting the mitotic progression of rapidly dividing cells by provoking the spindle assembly checkpoint, and eventually leading to the initiation of apoptosis (Prota et al., 2013). This is believed to be the major molecular mechanism for the anticancer activity of MTAs (Dumontet and Jordan, 2010; Zhao et al., 2016). However, one limitation of MTAs is that if cells arrested in mitosis do not die they could eventually return to the cell cycle. Thus, combination therapies to ensure that cells arrested in mitosis with MTAs end up dead could prove beneficial.

## Classical Plant-Derived MTAs

Because of the structural diversity of molecules they contain, plants are an ideal source of novel MTAs and lead compounds. Over the past decades, plant-derived MTAs have proved efficacious for the treatment of a wide spectrum of malignancies. Some of the earliest identified and most successful classes of MTAs are the taxanes and the vinca alkaloids.

### Taxanes

In the early 1960s, the National Cancer Institute launched a large-scale screening campaign to search for active anticancer compounds present in plant products, which led to the discovery of paclitaxel, the founding member of the taxane class (Cragg, 1998). The compound was originally isolated from crude extracts derived from the bark of the Pacific yew tree (*Taxus brevifolia Nutt*). Paclitaxel binds to the intermediate domain of β-tubulin within the interior lumen of microtubules (**Figures 1B,C**) (Wani et al., 1971). Binding of paclitaxel to microtubules results in a decrease in microtubule dynamics and arrests cells in mitosis. Although its structure and anticancer activity were discovered in the 1970s, limited availability from natural sources and low aqueous solubility hampered the drug’s development. It wasn’t until 1992 that paclitaxel was approved by the Food and Drug Administration to treat metastatic ovarian cancer. Currently, paclitaxel, alone or combined with other chemotherapeutic agents, has become one of the most commonly used chemotherapeutic agents and is indicated for the treatment of a wide range of cancers, including breast, ovarian, and lung cancers (**Table 1**).

**Table 1:** Plant-derived MTAs, their sources, and clinical indications.

Compound	Effect on microtubules	Original source	Other sources	Issues affecting development and clinical utility	Clinical indications
Taxanes Paclitaxel Docetaxel	Microtubule-stabilizing	*Taxus brevifolia Nutt*	*Corylus avellana* L., fermentation, plant cell culture	Low aqueous solubility; supply and formulation issues; side effects; resistance in some patients	Treatment of various cancers, including ovarian, breast, and lung cancers, melanoma, and Kaposi’s sarcoma
Vinca alkaloids Vinblastine Vincristine	Microtubule-destabilizing	*Catharanthus roseus G. Don.*	Exclusively present in the *Vinca* genus	Severe side effects, including thrombocytopenia and reversible peripheral neurotoxicity; resistance caused by changes in expression of tubulin isotypes and *P*-glycoprotein	Treatment of various cancers, including Hodgkin’s lymphoma, non-small cell lung cancer, bladder cancer, brain cancer, and testicular cancer
Colchicine	Microtubule-destabilizing	*Colchicum autumnale* L.	*Gloriosa superba* L.	Side effects, including gastrointestinal distress and neutropenia	Treatment of immune diseases, including gout, rheumatism, inflammation, and familial Mediterranean fever (FMF)
Combretastatin	Microtubule-destabilizing	*Combretum caffrum*	*Anogeissus leiocarpus, Guiera senegalensis, Quisqualis indica*	Poor pharmacokinetic properties due to high lipophilicity and low aqueous solubility	Treatment of anaplastic thyroid cancer, medullary thyroid cancer, and stage IV papillary or follicular thyroid cancer
Taccalonolides	Microtubule-stabilizing	*Tacca leontopetaloides*	Other *Tacca* species	Supply and formulation issues	Currently under investigation in a number of clinical trials
Taccabulin A	Microtubule-destabilizing	*Tacca chantrieri, Tacca integrifolia*	Other *Tacca* species	Not applicable	Currently under investigation in preclinical studies


In addition to *Taxus* species, *Corylus avellana* L. (hazel) has been reported to produce paclitaxel (Ottaggio et al., 2008). Interestingly, an endophytic fungus isolated from *Taxus* and other species was found to produce paclitaxel as well, suggesting that fermentation of microorganisms could serve as an alternative approach for paclitaxel production (Mirjalili et al., 2012; Somjaipeng et al., 2015). Plant cell cultures and fermentation of endophytic fungi have become an area of intensive investigation for the production of paclitaxel. In addition, the commercial successes and growing demand for paclitaxel sparked a search for new sources of taxanes. Docetaxel, a semi-synthetic analog of paclitaxel, has been approved to be used alone or with other drugs to treat cancers such as breast cancer, non-small cell lung cancer, and prostate cancer (**Table 1**). Docetaxel was synthesized from the natural precursor, 10-deacetylbaccatin III (Ojima et al., 1994). In contrast to the source of paclitaxel (the bark of *Taxus*), this precursor can be isolated from the needles of the European yew (*Taxus baccata*), allowing for improved availability due to its renewable source.

### Vinca Alkaloids

The vinca alkaloids comprise the second family of classical MTAs. These agents were first extracted from the leaves of the Madagascar periwinkle, *Catharanthus roseus G. Don.* (Apocynaceae) in the 1950s (Noble et al., 1958). Initially, the extracts were investigated for their effectiveness against diabetes, but failed to demonstrate activity. Surprisingly, however, promising activity was observed against lymphocytic leukemia in rats (Noble, 1990). Further investigation led to the discovery of vinblastine and vincristine. The vinca alkaloids bind a site located at the interface between tubulin heterodimers, termed the vinca-binding site (Moudi et al., 2013). At sub-stoichiometric concentrations, the vinca alkaloids associate with the high-affinity sites at the ends of microtubules, resulting in the suppression of microtubule assembly. However, at higher concentrations, the vinca alkaloids prefer to bind low-affinity, high capacity sites on unpolymerized β-tubulin, preventing the integration of tubulin into polymerized microtubules (**Figures 1B,C**).

The vinca alkaloids represent one of the most widely used chemotherapeutic drugs for various cancers, in particular for the treatment of hematological malignancies (**Table 1**). New clinical applications for the vinca alkaloids are being investigated, alone or in combination with other chemotherapeutic agents. The therapeutic efficacy of the vinca alkaloids has led to subsequent attempts to identify high alkaloid-producing plants, as well as investigation into production in cell suspension cultures. For example, III-Min Chung et al. (2011) screened 64 cultivars of *Catharanthus roseus* and found Cooler Rose Hot produced the highest concentration of serpentine alkaloids (Chung et al., 2011). Recently, culturing of endophytic fungi isolated from *Catharanthus roseus* has been explored as an alternative approach for the production of the vinca alkaloids (Kumar et al., 2013).

## Other Plant-Derived MTAs

In addition to the more familiar taxanes and vinca alkaloids, several other plant-derived MTAs with varying clinical utility have also been discovered, such as colchicine and combretastatin (**Table 1**).

### Colchicine

Colchicine is a compound first isolated from the bulbs and seeds of the autumn crocus (*Colchicum autumnale* L.). This agent binds the interface between α- and β-tubulin at a site distinct from the binding sites for the taxanes and the vinca alkaloids (**Figures 1B,C**). Colchicine is a potent microtubule-destabilizing agent that acts by suppressing the formation of lateral connections between protofilaments. In the clinic, this MTA has most commonly been used as an immunosuppressant for the treatment of various immune diseases, including gout, rheumatism, inflammation, and familial Mediterranean fever (Slobodnick et al., 2015). Colchicine is not approved for the treatment of neoplastic disease due to its severe toxicities (Stack et al., 2015). In recent years, *Gloriosa superba* L., an herbaceous climber with a wide distribution in tropical areas, has become another important plant source of colchicine.

### Combretastatin

Combretastatin binds the same site as colchicine and was originally extracted from the South African willow, *Combretum caffrum*. Combretastatin selectively suppresses tumor angiogenesis, a process crucial for cancer growth and metastasis. Importantly, combretastatin is not recognized by the ATP-dependent efflux transporters associated with drug resistance, making it a promising lead molecule for chemotherapy (Patil et al., 2015). This agent is currently being investigated for the treatment of several cancer types, such as anaplastic thyroid cancer and medullary thyroid cancer (**Table 1**) (Garon et al., 2016; Jaroch et al., 2016).

## Taccolonolides: A Novel Class of Plant-Derived MTAs

The first taccalonolide taccalin, an extremely bitter-tasting compound extracted from the tubers of *Tacca leontopetaloides*, was identified in the 1963. The complete structures of taccalonolides A and B from the rhizomes of *Tacca plantaginea* were solved in 1987. Cells treated with taccalonolides were found to exhibit a paclitaxel-like phenotype, and bioassay-guided fractionation subsequently identified taccalonolides A and E as microtubule-stabilizing agents (Tinley et al., 2003). Taccalonolides represent a novel class of MTAs because of their unique mechanism of action. Unlike the taxanes and other microtubule-stabilizing agents, taccalonolides A and E do not possess the ability to bind and polymerize tubulin *in vitro*, even in the presence of cytosolic cellular extracts.

To date, several other taccalonolides have been isolated and identified from *Tacca* species. For example, taccalonolides A-M and W-Y were isolated from *Tacca plantaginea*, taccalonolides N and R-V were isolated from *Tacca paxiana*, and taccalonolides O-Q were isolated from *Tacca subflabellata* (Li et al., 2014). In addition, a group of rare taccalonolides, including taccalonolides AA, AB, R, T, and Z, were isolated from *Tacca chantrieri* and *Tacca integrifolia* (Peng et al., 2011). Taccalonolide AF, a promising molecule isolated from *Tacca plantaginea*, covalently binds β-tubulin (**Figure 1C**) and thereby imposes an unparalleled level of microtubule stability (Li et al., 2011). Its distinct anticancer activity is further strengthened by its lack of recognition by ATP-dependent efflux transporters. Together, these features give taccalonolide AF the ability to circumvent innate and acquired resistance mechanisms caused by mutations in the taxane binding site and exert persistent cellular effects due to the lack of efflux. Taccalonolides are currently under investigation in a number of clinical trials.

Taccabulin A, a cytotoxic retro-dihydrochalcone also isolated from *Tacca* species, was identified by a stringent, bioassay-guided fractionation procedure designed to isolate rare and potent taccalonolides. Mechanistic investigation revealed that Taccabulin A binds to the colchicine binding site on tubulin, thereby eliciting microtubule depolymerization. Interestingly, taccabulin A has the ability to overcome resistance mechanisms mediated by ATP-dependent efflux transporters and βIII-tubulin (Risinger et al., 2013).

## Conclusion and Perspectives

The great successes of the taxanes and the vinca alkaloids, as well as a subset of candidates exhibiting promising efficacy in clinical trials, make microtubules a continuing attractive target for cancer therapeutics. Even for MTAs that are not used as anticancer drugs, such as colchicine and nocodazole, they are important tools for studying microtubule structures and functions, thus facilitating, in an indirect way, the discovery of anticancer drugs.

Limitations of the current spectrum of MTAs, which include the development of innate and acquired resistance and intolerable toxicities, demonstrate the need for the discovery of new MTAs (Dumontet and Jordan, 2010; Xie et al., 2016). The structures of MTAs produced by plants are quite different, ranging from simple bicyclics, like combretastatins, to complex molecules, like taccalonolides (Sackett and Fojo, 2011). The structural diversity of MTAs suggests that they may serve as an ideal source of novel lead compounds that exhibit microtubule-disrupting activities. The ideal features of novel MTAs include robust anticancer activity, low toxicity to normal tissues, and circumventing resistance to current MTAs. Given the promising profiles of taccalonolides, traditional medicinal plants widely used for a variety of ailments, such as the genus *Tacca*, might be targeted for screening novel MTAs in the future.

Currently, relatively little of the world’s plant biodiversity has been tapped in the search for MTAs, presenting a major opportunity for the discovery of novel bioactive compounds from plants. However, several perceived drawbacks challenge the interest in searching for MTAs from plants. These include issues of supply and access, difficulties in high-throughput screening due to the complexities of plant extracts, and the extremely laborious effort and high cost of generating plant collections. Fortunately, as a result of recent advances in synthetic methodologies and genetic engineering techniques, it is now feasible to circumvent challenges of supply using total-or semi-synthetic methods and *in vitro* culture approaches.

In addition, screening from crude extracts and pre-fractionated extracts has become more convenient than ever due to improvements in fractionation methods that can more easily isolate and purify biologically active compounds, as well as the development of analytic techniques capable of elucidating compound structures. Attempts to generate pure compound libraries prior to biological profiling are also an alternative and effective approach for natural product screening. Development of sensitive, high-throughput bioassays to identify less abundant active compounds from extract mixtures should be prioritized to maximize yield. Finally, designing strategies to efficiently and effectively access plant biodiversity is an important topic for future discussion, and establishment of appropriate frameworks for international collaborations would greatly benefit access to the Earth’s biodiversity and would undoubtedly facilitate the discovery of novel MTAs.

## Author Contributions

All authors listed, have made substantial, direct and intellectual contribution to the work, and approved it for publication.

## Conflict of Interest Statement

The authors declare that the research was conducted in the absence of any commercial or financial relationships that could be construed as a potential conflict of interest.
